# Genes Contributing to Domestication of Rice Seed Traits and Its Global Expansion

**DOI:** 10.3390/genes9100489

**Published:** 2018-10-10

**Authors:** Haiyang Liu, Qiuping Li, Yongzhong Xing

**Affiliations:** 1 National Key Laboratory of Crop Genetic Improvement and National Center of Plant Gene Research (Wuhan), Huazhong Agricultural University, Wuhan 430070, China; daydayup@webmail.hzau.edu.cn (H.L.); liqiuping@webmail.hzau.edu.cn (Q.L.); 2 Wuhan Life Origin Biotech Joint Stock Co., Ltd., Wuhan 430206, China

**Keywords:** domestication, seed shattering, seed dormancy, flowering time, global expansion, parallel evolution

## Abstract

Asian rice (*Oryza sativa*) and African rice (*Oryza glaberrima*) are separately domesticated from their wild ancestors *Oryza rufipogon* and *Oryza barthii*, which are very sensitive to daylength. In the process of domestication, some traits that are favorable for the natural survival of wild rice such as seed dormancy and shattering have become favorable ones for human consumption due to the loss-of-function mutations in the genes that are underlying these traits. As a consequence, many genes that are related to these kinds of traits have been fixed with favorable alleles in modern cultivars by artificial selection. After domestication, *Oryza sativa* cultivars gradually spread to temperate and cool regions from the tropics and subtropics due to the loss of their photoperiod sensitivity. In this paper, we review the characteristics of domestication-related seed traits and heading dates in rice, including the key genes controlling these traits, the differences in allelic diversity between wild rice and cultivars, the geographic distribution of alleles, and the regulatory pathways of these traits. A comprehensive comparison shows that these genes contributed to rice domestication and its global expansion. In addition, these traits have also experienced parallel evolution by artificial selection on the homologues of key genes in other cereals.

## 1. Introduction

A suite of common traits is selected during the domestication of crops, which are collectively known as a “domestication syndrome”. These traits make domesticated species different from their wild ancestors. In cereals, these traits include morphological traits such as larger grains, a loss of seed dispersal, increased apical dominance, more determinate growth, and physiological traits including seed dormancy, seed shattering, bitter substances in edible structures, photoperiod sensitivity, and synchronized flowering [1]. The seed-related traits of dormancy, seed shattering, and grain size determine which kinds of grain humans can choose for domestication, and the photoperiod-sensitive heading date limits the optimal growing region of rice. Rice is a model crop plant due to its small genome size, high-quality genome sequence, and efficient transformation [2]. In the last two decades, many genes that are related to domestication and global expansion have been isolated in rice. In this review, we summarize the genes controlling seed dormancy, seed shattering, grain size, and flowering time, which contribute to rice domestication and its global expansion. In addition, the homologues in other crops are also discussed to verify parallel evolution during domestication.

## 2. Seed Shattering in Rice

Wild rice seeds fall off after maturity to ensure their natural propagation in the natural environment. However, seed shattering causes yield loss for domesticated crop plants during harvest. The non-shattering trait is likely to undergo strong selection early in domestication and further facilitate the fixation of other domestication characters. Hence, the loss of seed shattering is considered to be direct ecological evidence for wild rice domestication.

### 2.1. Seed Shattering Genes Identified from Natural Variation

In rice, seed shattering is regulated by the formation of an abscission zone (AZ), which is composed of several layers of small and dense cytoplasmic cells in the joint between the lemma and pedicel [3]. Several shattering quantitative trait loci (QTLs) have been identified including *SH4*, encoding an Myb3 transcription factor, and *qSH1*, encoding a BELL1-like homeodomain protein (Table 1) [4,5,6]. The mutated alleles *qsh1* and *sh4* cause seed non-shattering owing to the absence of abscission layer formation (Table 1). The *SHAT1* gene, which encodes an APETALA2 transcription factor, is identified from induced mutant through a 60Co γ-ray radiation and is required for seed shattering through specifying AZ development in rice. The expression of *SHAT1* in the abscission layer is positively regulated by *SH4*, whose mutation results in incomplete development and the partial functioning of the AZ. *qSH1* functions downstream of *SHAT1* and *SH4*, and is involved in a positive feedback loop with *SHAT1* and *SH4* through the maintenance of *SHAT1* and *SH4* expression in the AZ, thereby promoting AZ differentiation [7].

### 2.2. Mutated Alleles Led to Seed Non-Shattering Domestication

*Sh4* was detected using an F2 population derived from an *indica*-type cultivar and the wild rice progenitor *Oryza nivara* (an annual form of *Oryza rufipogon*). The nucleotide substitution guanine/ thymine (G/T) in the first exon of *SH4* results in a mutated allele carried by all of the cultivars compared with the alleles in wild rice. The *SH4* phylogeny, together with neutrality tests and coalescent simulations, suggested that *sh4* had a single origin and was fixed to have a mutated allele by artificial selection during domestication [4,5,19], indicating its vital role in rice domestication. However, a near isogenic line carrying *sh4* alleles showed strong seed-shattering behavior in the wild rice genetic background and weedy rice, indicating that other genes redundantly regulate abscission layer formation [20,21,22,23]. The interaction between the *sh4* and *qSH3* loci inhibits the formation of the abscission layer in rice [20,21,22,24,25]. The effect of *qSH3* on seed shattering was weaker than that of *qSH1* or *sh4* when evaluated in the genetic background of cultivated rice [24,25]. *qSH1* was detected using a mapping population derived from the *japonica* cultivar Nipponbare and *indica* cultivar Kasalath. The single nucleotide polymorphism (SNP) (G/T) in the 5’ regulatory region of *qSH1* was conserved in *japonica* and highly associated with shattering in the temperate *japonica* subspecies, and has not been introgressed into *indica* subspecies [6,19]. 

### 2.3. The Parallel Evolution of the Non-Shattering Trait in Cereal Crops

Some seed shattering genes are identified from Asian rice including *SH4* and *qSH1*, and some other genes are identified in African rice, including *GL4* and *ObSH3* [4,5,6,26,27]. *GL4*, encoding an Myb3 transcription factor, was identified in *Oryza barthii*, which is the ancestor of African cultivars (*Oryza. glaberrima*). A cytosine/thymine (C/T) substitution in the *GL4* gene resulted in a premature stop codon and led to small seeds and the loss of seed shattering [26]. *ObSH3*, encoding a YABBY transcription factor, was identified from an F_2_ population derived from a cross between the African wild rice accession W1411 and the non-shattering African cultivar IRGC104165. Most accessions with both mutations *gl4* and *obsh3* occur in arid regions north of 11° N in African [26,27]. Their orthologues in either African rice or Asian rice have the conserved functions and have been subjected to selection. For example, *OgSH1* and *OgSH4*, the orthologs of the Asian rice-shattering genes *OsSh1* and *SH4*, also play important roles in controlling seed shattering in African rice. *Ossh4* and *Ogsh4* were selected in parallel during the domestication of African and Asian rice, respectively [4,5,27,28]. Therefore, artificial selection on common shattering genes resulted in non-shattering in Asian and African cultivars. Meanwhile, seed non-shattering due to the loss of the abscission layer also exists in other crops. *Sh1*, a homologue of *OsSH1* in sorghum, also regulates seed shattering [29]; *ZmSh1-1*, *ZmSh1-5.1*, and *ZmSh1-5.2*, the homologues of *ObSH3* in maize, have been verified to control seed shattering by QTL mapping with a large mapping population [27,29]. The *Q* gene, the homologue of *SHAT1* in wheat, encodes a member of the APETALA2 family of transcription factors and confers the free-threshing character [30,31]. The same regulatory point mutation in rice *Sh1* was also identified in an *Sh1* orthologue gene in *Brassica*, and is responsible for the seed dispersal structures produced by natural selection [32]. Taken together, these findings indicate that parallel selection on non-shattering exists during sorghum, rice, and maize domestications.

## 3. Seed Dormancy in Rice

Seed dormancy is a special period in the plant life cycle when seed germination is unable to proceed under a combination of environmental factors that are normally suitable for the germination of non-dormant seeds. Dormancy is a mechanism in wild species to prevent germination during unsuitable external conditions. Excessive seed dormancy is not a desired trait in crops. Some degree of dormancy is desired to prevent pre-harvest sprouting. Hence, domesticated crops have undergone selection against dormancy. Abscisic acid (ABA) and gibberellin (GA) are two major regulators of seed dormancy and germination. Abscisic acid positively regulates dormancy induction and maintenance, while GA promotes seed germination [33,34].

### 3.1. Seed Dormancy Genes Identified from Natural Variation

Many QTLs affecting seed dormancy or germination-related traits have been mapped in rice [35,36,37,38,39,40,41], but only a few QTLs have been cloned in rice (Table 1), such as *Sdr4*, encoding a novel protein; *qSD7-1/Rc*, encoding a bHLH transcription factor; and *qSD1-2*, encoding OsGA20ox2 [8,9,10,42]. *Sdr4* acts as a seed dormancy-specific regulator that is under the control of *OsVP1*, which is a global positive regulator of seed maturation through its effect on ABA signaling and the regulation of the expression of *OsDOG1L-1*, which is a positive regulator of dormancy in rice [8]. *qSD7-1/Rc* controls dormancy and pigment traits by regulating ABA and the flavonoid biosynthetic pathways, respectively [9]. However, *qSD1-2* regulates seed dormancy by controlling the seed GA level [10]. A recent study demonstrated that the antagonistic relationship between the ABA and GA metabolic pathways regulates the switch of cereal seeds between dormancy and germination [43].

### 3.2. Mutated Alleles Led to Seed Dormancy Domestication 

The 18-bp (base pair) direct repeat sequence variation resulting from a double-strand cleavage and repair event in *Sdr4* substantially contributes to the differences in seed dormancy between *japonica* (Nipponbare) and *indica* (Kasalath) cultivars. A sequence analysis of 59 cultivars and 46 accessions of *O. rufipogon* revealed that the *Sdr4-n* sequence that causes reduced dormancy was not found in any wild rice accessions, and that the *Sdr4-n* in *indica* cultivars is introgressed from japonica rice. *Sdr4-n* appears to have been produced through at least two mutation events from the closest *O. rufipogon* allele among the examined accessions. *Sdr4-k* and *Sdr4-k′* in *indica* were inherited from these subgroups of the wild ancestor [8]. *qSD7-1/qPC7* controls the seed dormancy/pericarp color in weedy red rice. The dormancy-enhancing alleles *qSD7-1/qPC7* was differentiated into two groups that are generally associated with the tropical and temperate ecotypes of weedy rice. *qSD7-1/qPC7* may contribute the most to weed adaptation [9]. A loss-of-function mutation in *qSD1-2* enhances seed dormancy and results in semi-dwarfism, which has been used to develop high-yield, semi-dwarf varieties worldwide. The *sd1* mutant originally occurred in an *O. rufipogon* population and in weedy rice. The allelic distribution of *qSD1-2/OsGA20ox2* was found to be associated with the subspeciation of *indica* and *japonica* rice [44]. However, there is no evidence that the primitive *indica*-specific and *japonica*-specific alleles are functionally differentiated at the presumably domestication-related locus of *qSD1-2/OsGA20ox2* [35].

### 3.3. The Parallel Evolution of Seed Dormancy in Cereals

Recently, many QTLs affecting seed dormancy or germination-related traits have been identified in plant species such as barley [45,46,47] and wheat [40,48,49,50]. However, most seed dormancy QTLs have not been finely mapped. Currently, it is difficult to infer the identities of these QTLs. Based on synteny analysis, *HvGA20ox* has been suggested as the candidate of a seed dormancy QTL in barley [47]. The orthologues of *OsSdr4* in wheat, namely *TaSdr*, in which a single-nucleotide mutation causes a distinct phenotype in seed germination, have been demonstrated to be key regulators of pre-harvest sprouting in wheat [51]. Seed dormancy genes are probably under parallel selection in cereals, which needs to be further demonstrated by testing the function of more homologues of seed dormancy genes in other cereal crops in the future.

## 4. Grain Size in Rice

Plant seeds are major sources of human nutrition and are the major means of crop propagation. The grain size of wild rice is significantly smaller than that of cultivated rice [1]. Early in domestication, farmers preferred to select larger seeds to increase grain yield and obtain more food during domestication [52]. Therefore, seed size has always been subjected to selection.

### 4.1. Grain Size Genes Associated with Domestication in Rice 

Dozens of major QTLs for grain size have been molecularly characterized, and their regulatory roles in determining grain size or weight have been explored. The beneficial alleles *gs3* [11,12], *gw5* [15,16,17,18], *GW8* [53,54], *gif1* [55], *GLW7* [56], *qLGY3/OsLG3b* [13,14,57], *GL7/GW7/SLG7* [54,58,59], and *gl4* [26] were artificially selected in modern rice varieties during domestication. However, the beneficial alleles *qgl3.1* [60,61,62], *tgw6* [63], *GS2/GL2/OsGRF4/PT2* [64,65,66,67,68], *GS5* [69], *GW6a* [70], *tgw3* [71,72,73], and *GS9* [74] are rare alleles, which would have mutated recently, and thus were not widely selected by breeders due to limited time.

### 4.2. Mutated Alleles Contributing to Domestication 

*GS3* contains four putative domains, and the organ size regulation (OSR) domain is necessary and sufficient for its function as a negative regulator [11,12]. *GS3* has undergone positive selection in cultivars [75,76]. A cytosine/adenine (C/A) mutation in the second exon is associated with large grain size and classifies global rice collections into long-grain and short-grain groups. C/A variation exists in the wild rice gene bank [75,77,78], and the C/A variation in wild rice may have resulted from the gene flow from cultivated rice. However, this mutation does not play a similar role in wild rice [77]. The short-grain alleles of *GS3* have multiple independent origins, because farmers and early breeders imposed artificial selection, favoring short seeds [79]. The long-grain allele *gs3* likely originated from a *japonica*-like ancestor, and was subsequently introduced into *indica* by introgression [77]. However, a haplotype network and phylogenetic analyses showed that the *japonica* and *indica* haplotypes evolved independently [80].

*qLGY3/OsLG3b* encodes the MADS-domain transcription factor OsMADS1, and it regulates grain size by interacting with the Gγ subunits GS3 and DEP1. Six SNPs in the *OsLG3b* region led to alternative splicing, which was associated with grain length, resulting in increases in both the grain quality and yield potential of rice [13,14]. Haplotype analysis revealed that the long-grain allele of *OsLG3b* might have arisen after the domestication of *tropical japonica*, and then spread to the subspecies *indica* or *temperate japonica* by natural crossing and artificial selection, which is similar to the events related to the *GS3* gene that lead to the improvement of *tropical japonica* [14]. 

*GW5/qSW5* encodes a plasma membrane-associated protein with IQ calmodulin-binding motifs, and is a novel positive regulator of brassinosteroid (BR) signaling that controls grain width and weight through the proteasomal degradation pathway [15,16,17,18]. A 1212-bp deletion (DEL2) in *japonica* varieties and a 950-bp deletion (DEL1) in *indica* varieties in the promoter region of *qSW5* are confirmed to be the causal mutations that led to increases in both grain width and grain weight, and have strong correlations with grain width in rice. Both the DEL1 and DEL2 deletions likely originated in different wild rice accessions during rice domestication, and were enriched by artificial selection, as well as the propagation of cultivation and natural crosses, and eventually became widely utilized by rice breeders [15,16,17,18]. A nucleotide diversity analysis showed that *qSW5* has high nucleic acid polymorphism in both cultivated and wild rice populations, and it has been subjected to positive selection for genetic improvement [81]. 

### 4.3. The Parallel Evolution of Grain Size in Cereals

The *GS3*-homologous gene *ZmGS3* in maize contains the same conserved domain as *GS3*. Correlation analysis shows that the SNP located in the fifth exon is significantly correlated with the kennel length of maize [82]. The *TaGW2-6A* of a *GW2* homologue in wheat and the *ZmGW2-CHR4* and *ZmGW2-CHR5* of *GW2* homologues in maize are highly associated with grain width and grain weight in a large germplasm collection [83,84]. The orthologue of rice *GS5* in wheat, *TaGS5-3A-T*, is significantly associated with larger grain size and a higher thousand-kernel weight [85]. In contrast to the evolutionary model of *GS5* in rice, *TaGS5-3A-T* is positively selected in Chinese wheat breeding, and undergoes selection during each polyploidization event [85,86]. Among the 15 seed size genes previously identified to be under selection in rice or maize, 12 orthologues in sorghum have been under selection during domestication [87]. The major grain size genes in rice and their homologues in other cereal crops have large effects on grain weight; therefore, they have been easily selected based on trait performance. Therefore, grain size has undergone parallel evolution in cereals.

## 5. Flowering and Adaptation in Rice Expansion

Rice flowering time is determined by internal genetic factors and external environmental factors such as daylength, temperature, drought, nutrients, and biotic stresses. The molecular mechanism of rice flowering has been well characterized and has been recently reviewed [88]. Many genes are included in the review, but here we mainly focused on the genes that have been isolated from natural variations and are related to rice adaptation. *O. rufipogon* is mainly in a limited tropical region nearby and has been domesticated to the cultivar *O. sativa* in the Yangtze River valley region in China [78,89]. After a long process of domestication and improvement, rice has been successfully grown worldwide as a global crop, indicating that the rich variation in heading date genes has allowed cultivated rice to adapt to different environments, especially the changing daylength at different latitudes [90]. Rice is a short-day plant. Rice flowering is repressed when the daylength is longer than 13.5 h [91]. In tropical regions, the daylength is less than 13.5 h, and the daily temperature is high, which ensures that rice can grow all year. However, in other regions, such as temperate regions, rice only grows in the short and warm summer, when the daylength is long. Therefore, during rice expansion, cultivars have gradually adapted to the long daylength at high latitudes. Here, we will review the genes that are responsible for rice adaptation to various daylengths, trace the nucleotide changes, and try to reveal how cultivars expanded into diverse regions.

### 5.1. Heading Date Genes Identified from Natural Variation

Cultivars have abundant variation in flowering time [92], indicating that many heading date genes are responsible for rice flowering. Dozens of genes have been mapped for heading date in rice [93]. Eighteen QTLs (Hd1-Hd18) were detected with the different populations that have been derived from crosses between Nipponbare and Kasalath and between Koshihikari and Hayamasari, and most of the QTLs have been cloned [94,95,96,97]. *Hd1*, the first cloned flowering gene from natural variation in rice, is the orthologue of *CONSTANT*, which encodes a transcription factor with a zinc-finger domain and a CONSTANS, CO-like, and TOC1 (CCT) domain. This gene is a bifunctional regulator that promotes flowering in short days and delays flowering in long days [98]. *Hd2*, which is also named *Ghd7.1/OsPRR37/DTH7*, encodes a transcription factor with a pseudoreceiver domain and a CCT domain. This gene delays flowering under long days [99,100,101]. *qHd3* includes two heading date genes, namely *Hd3a* and *Hd3b* (*Hd17*/*OsELF3*/*EF7*) [102,103,104]. *Hd3a* encodes one of the mobile flowering signal florigens in rice, and *RFT1* encodes another florigen. Both genes belong to the phosphatidylethanolamine-binding protein (PEBP) gene family [105,106,107,108]. *Hd3b*, which is also named *OsELF3*, *Hd17*, and *EF7*, is the homologue of *ELF3*, which is a component of the *Arabidopsis* circadian clock [104]. *Hd4*/*Ghd7*, encoding a transcription factor with a CCT domain, delays flowering on long days [109]. *Hd5*/*Ghd8*/*DTH8*/*LHD1* is a member of the heme-associated proteins 3 (HAP3) family [110,111,112]. *Hd6* and *Hd16*/*EL1/EF7* encode the subunits of casein kinase II and casein kinase I separately [113,114,115]. *Hd9/DTH3/OsMADS50*, the homologue of *SOC1* in rice, is a member of the MADS-box gene family [116]. *Hd18* encodes an amine oxidase domain-containing protein and is the homologue of *Arabidopsis* flowering locus D [97]. With other bi-parentally derived populations, additional heading date QTLs have been identified and cloned such as *Ehd1*, *DTH2*, and *Ehd4* [117,118,119]. *Ehd1* encodes a B-type response regulator and promotes flowering independently of *Hd1*. *DTH2* also encodes a transcription factor with a zinc-finger domain and CCT domain, and delays flowering on long days. *Ehd4* encodes a CCCH-type zinc finger transcription factor and promotes flowering. Interestingly, when QTL analyses were performed using F2 populations from crosses between Koshihikari and 12 cultivars originating from various regions in Asia, most of the major QTLs were identified, including *Hd1*, *Hd6*, *Hd16*, *Hd17*, *Ghd7*, *Ghd7.1*, *Ghd8*, *Hd3a*, and *RFT1* [120,121,122]. In addition, many of the minor QTLs were identified with advanced populations [122]. Genome-wide association analysis studies on the basis of diverse germplasm collections identified some of the flowering genes that were detected in bi-parental mapping populations [123,124,125]. These results strongly suggest that these genes largely determine the variations in the heading date of rice.

### 5.2. Regulatory Networks of Flowering

**Figure 1:**
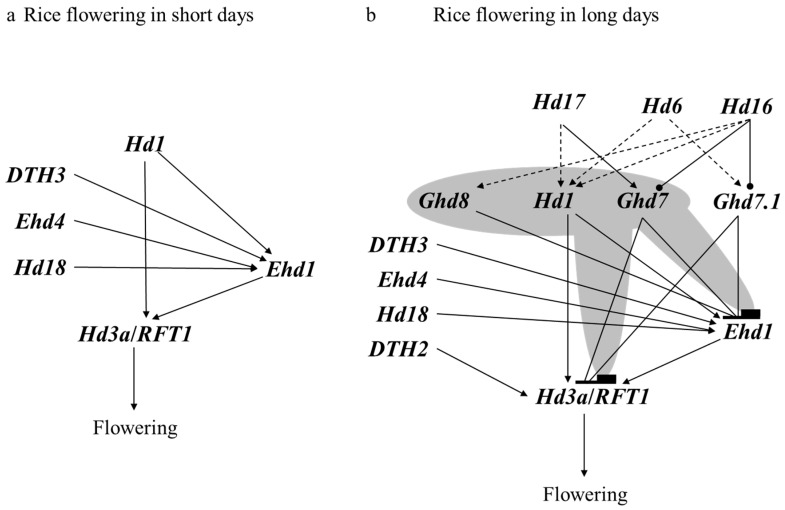
The regulatory network of rice flowering. Lines with a triangle indicate active transcriptional regulation; lines with a cap indicate a repression of transcriptional regulation; lines with a dot indicate phosphorylation; dotted lines indicate genetic interaction; and the dark background indicates that *Hd1* interacts with *Ghd8* and *Ghd7* to largely repress *Ehd1* and *Hd3a*/*RFT1* expression.

Combined with the latest research findings, a draft regulatory network of how these QTLs/genes coordinate to determine rice flowering time is suggested (Figure 1). The key regulators of flowering are the florigens Hd3a and RFT1, which are expressed in leaves, and then move to the shoot to accelerate flowering [105,106,107,108]. *Hd1* only triggers the expression of florigen genes directly in leaves, but it can switch to repress the expression of florigen genes when *Ghd7* and *Ghd8* are present in long days [126,127,128,129,130]. *Ehd1* also triggers the expression of florigen genes independently of *Hd1*; moreover, it is regulated by *Ghd7*, *Ghd8*, *Ghd7.1*, *Ehd4*, *DTH2*, *DTH3*, and other flowering genes [101,109,111,112,116,118]. In addition, other regulators that are dependent on these major genes have been identified as contributing to flowering. *Hd17* is a component of the circadian clock that delays flowering, depending on *Hd1* [104]. *Hd6* and *Hd16* encode kinase and directly phosphorylate GHD7 and GHD7.1, and interact genetically with *Ghd7*, *Ghd7.1*, *Ghd8*, and *Hd1* to further delay flowering [113,114,131]. Even more extreme, there are two combinations that lead to non-flowering in natural long days. One is the combination of functional *Hd1*, *Ghd7*, and *Ghd8* in Zhenshan97, while another includes non-functional *ehd1* and *rft1* in both the Nona Bokra background and a recombinant inbred line derived from Guangluai 4 and Taichung 65 [126,132,133]. Taken together, these studies show that cultivars with different combinations of known flowering genes can exhibit extensive variation in the heading date, from earlier flowering to extremely later flowering or even non-flowering on long days. On short days, only a few genes such as *Hd1*, *Ehd4*, *DTH3*, and *Hd18* still promote flowering as they do on long days, while most others have weak effects or even no effect such as *Ghd7*, *Ghd7.1*, *Ghd8*, *Hd17*, and *DTH2* [100,104,109,111,116,117,118,127,128,129].

### 5.3. Diverse Alleles of Flowering Genes in Wild Rice and Cultivars 

*Oryza rufipogon* has limited genetic and nucleotide diversity for a partial outcrossing species [89,134]. Here, we summarize the functional diversities of known flowering genes in wild rice and cultivars (Table 2). Some key flowering genes have already diversified into functional (strong) and non-functional alleles in wild rice such as *Hd1*, *Hd6*, *Hd16*, and *RFT1*, and some genes such as *Ehd4, Hd17*, and *Hd3a* have generated weak alleles. However, no non-functional alleles have been identified for the strong photoperiod sensitivity genes *Ghd7*, *Ghd7.1*, *Ghd8*, *DTH2*, and *DTH3* in wild rice. These results indicate that the functional mutated alleles of some flowering genes exist in wild rice, but only in rare wild rice accessions. In cultivars, all of the flowering genes have several kinds of mutant alleles with a weak effect or no effect, in addition to the pre-existing alleles in wild rice. The newly generated alleles are enriched in high-latitude regions, because they are artificially selected to be grown under long-day conditions due to their weak or lack of photoperiod sensitivity [104,117]. Both *Ghd7* and *Ghd7.1* are sensitive to photoperiod. The alleles with strong effects are preserved from wild rice, but the *indica* and *japonica* alleles independently originated from different wild rice accessions [100,135], which indicates that *indica*–*japonica* differentiation has already occurred in wild rice. This notion is also supported by an evolutionary analysis of a major reproductive barrier regulator, *S5* [136]. Weak alleles or non-functional alleles of *Ghd7* and *Ghd7.1* were then generated in *japonica* and *indica* in parallel. Thus, the retention of their pre-existing genetic variants in ancestral species and the acquisition of mutations after domestication have increased the natural variation in the heading date.

An analysis of the eco-geographical distribution patterns of flowering genes showed that flowering repressors are present in low-latitude regions at high frequencies, while the activators (except *Hd1*) are present in high-latitude regions at high frequencies. As an important flowering signal integrator, the exception—*Hd1*—has an eco-geographical distribution pattern that is well explained by its bifunctionality. When *Hd1* is combined with the non-functional alleles *ghd7* and *ghd8*, *Hd1* promotes flowering under either long-day or short-day conditions [128]. When *Hd1* is combined with *Ghd7* and *Ghd8*, Hd1 interacts with the repressors of Ghd7 and Ghd8 under long-day conditions, and in turn greatly delays the flowering of this genotype, which is mainly grown in tropical regions. In addition, the interactions between *Hd6*, *Hd16*, and *Ghd7*, *Ghd8*, *Ghd7.1*, *Hd1* significantly increase the effects of these genes on the heading date [114,137,138]. These genes likely work together and may be the components of a large complex. Therefore, these interactions should be taken into consideration when developing a cultivar for a local region. With the help of humans/breeders, cultivars with different gene combinations are grown in optimized ecological regions, including high-latitude and low-latitude regions, to generate the maximum rice product [126]. However, for the flowering activators such as *RFT1*, the defective allele would be limited to the tropical region, and functional alleles would move to northern regions or the regions with double-cropping seasons such as those where early rice is grown, where the daylength is long throughout the cropping season [132,133]. Similarly, the functional *Hd18* with a minor effect is present in Northeastern China at a higher frequency [139].

### 5.4. The Parallel Evolution of Key Flowering Genes in Cereals

Plants are classified into three types according to their responses to photoperiods: short-day plants such as rice, maize, and sorghum; long-day plants such as wheat and barley; and day-neutral plants such as tomato [141]. When grown under the same daylength conditions, short-day and long-day plants show opposite responses. However, accessions with different photoperiod response-related genes exhibit various photoperiod responses. We have described how Asian rice expands into different latitudes from its limited region of origin. The other crops such as wheat, maize, and sorghum also adapt to local photoperiod conditions and consequently expand to different latitudes.

The major determinant of long-day response in barley has been cloned as *photoperiod-H1* (*Ppd-H1*) in a colinear region of *Ghd7.1*. In spring barley, an amino acid change in the CCT domain caused a reduced photoperiod response, which extended the growth period of barley, allowing it to adapt to the long growing seasons and produce higher yields in Western Europe and North America [142]. Different mutations in *SbPRR37*, the *Ghd7.1* homologue in sorghum, also reduce the photoperiod sensitivity and cause sorghum to flower earlier, which is critical for the cultivation of this tropical crop in temperate regions worldwide [143]. *BvBTC1*, the homologue of *Ghd7.1* in sugar beet (*Beta vulgaris*), is a master switch distinguishing annual from biennial. The loss-of-function *BvBTC1* allele confers a reduced photoperiod sensitivity, later flowering, and bienniality [144]. Taken together, these findings indicate that *Ghd7.1* and its homologues are key factors in expanding the cultivation of cereals. However, no identical mutant alleles were found in different crops, indicating that mutations occurred after species differentiation, but not from the common ancestor. *Ghd7* is another important major determinant of photoperiod response and adaptation in rice. *ZmCCT9* and *ZmCCT10* are homologues of *Ghd7* in maize. Insertions of transposable elements in the promoters of both genes caused a change in mRNA expression, and thus contributed to maize adaptation to higher latitudes after maize was domesticated from southern Mexico [145]. Mutations of *EAM8*, the homologue of *Hd17*, facilitate adaptation to a short growing season in barley and expand the geographic range of this species [146]. In sorghum, besides *Ma1*/*SbPRR37* and *EAM8*, *Ma6*, which is the homologue of *Ghd7*, and *FT*, which is the homologue of *Hd3a* and *RFT1* in sorghum, also regulates photoperiod flowering, and collectively with other genes contribute to its adaptation to diverse environments [147]. In addition, the parallel domestication of *Hd1* was elucidated in sorghum, foxtail millet, and rice [148]. Thus, the photoperiod response genes *Ghd7.1, Ghd7, Hd1, ELF3, Hd3a*, and their homologues underwent parallel selection, and the loss of function of these genes helped crops extend their ranges into higher latitudes from the original range in the tropics or subtropics.

## 6. Conclusions

**Figure 2:**
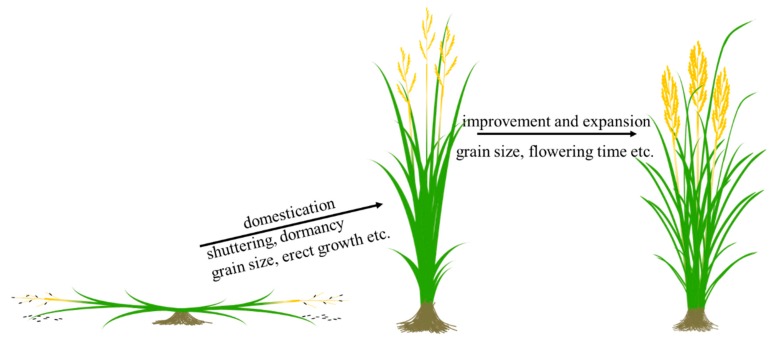
Two-step selection from wild rice to modern cultivars.

As described above, many important genes contribute to domestication in rice by loss of function. Moreover, many homologues in other cereals have conserved functions, which indicates that parallel evolution plays an important role in the “domestication syndrome”. Parallel evolution provides us with the chance to examine the function of homologues in other cereals once a major gene for such traits has been identified. In general, domestication syndrome genes are divided into two types according to their distribution in cultivars and wild rice. The genes of type one control prostrate growth (not included in this review), seed dormancy, and seed shattering. Loss-of-function mutations in these genes are required for rice domestication, because these mutations allow cultivars to grow more easily and produce more grains initially. In contrast, the genes of type two have diverse functional alleles in cultivars, but some strong functional alleles in cultivars come directly from wild rice, and weak or non-functional alleles are generated in cultivars such as genes for grain size and flowering time. These genes contribute to rice domestication but play more important roles in rice global expansion and genetic improvement to develop modern rice. In brief, the domestication of wild traits such as prostrate growth, seed dormancy, and seed shattering is the sudden result of loss-of-function mutations, and adaptive traits determining rice expansion such as photoperiod sensitivity are the continuing results of the mutations from strong alleles to weak alleles, and then to non-functional alleles (Figure 2). 

## Figures and Tables

**Table 1:** Reported genes of domestication traits including seed shattering, seed dormancy, and grain size.

Gene	MSU_LOC	RAP_LOC	Protein Category	Causative Mutation	References
*SH4*	LOC_Os04g57530	Os04g0670900	Myb-like transcription factor	G/T substitution in exon	[4,5]
*qSH1*	LOC_Os01g62920	Os01g0848400	BEL1-type transcription factor	G/T substitution in the 5′ UTR	[6]
*Sdr4*	LOC_Os07g39700	Os07g0585700	Novel protein	18-bp direct repeat	[8]
*qSD7-1/qPC7*	LOC_Os07g11020	Os07g0211500	bHLH transcription factor	14-bp deletion in exon	[9]
*qSD1-2*	LOC_Os01g66100	Os01g0883800	Gibberellin-20 oxidase	382-bp deletion	[10]
*GS3*		Os03g0407400	G protein γ subunit	C/A substitution in exon	[11,12]
*qLGY3/OsLG3b*	LOC_Os03g11614	Os03g0215400	MADS transcription factor	Six SNPs in exon	[13,14]
*GW5*/*qSW5*	LOC_Os05g09520	Os05g0187500	Plasma membrane	1212-bp or 950-bp deletion in the promoter	[15,16,17,18]

Bp: Base pair, SNP: Single nucleotide polymorphism; G/T: G substitutes for T; C/A: C substitutes for A; Myb-like: Myeloblastosis like; bHLH: Basic helix-loop-helix; MADS: MCM1, AGAMOUS, DEFICIENS, SRF.

**Table 2:** Functional and non-functional alleles of flowering genes in cultivars and wild rice.

Gene Name	MSU_LOC	RAP_LOC	Cultivars	Wild Rice
*DTH2*	LOC_Os02g49230	Os02g0724000	F/N	F [117]
*Ehd4*	LOC_Os03g02160	Os03g0112700	F/W	F/W [118]
*DTH3/OsMADS50*	LOC_Os03g03070	Os03g0122600	F/N	F [116]
*Hd6*	LOC_Os03g55389	Os03g0762000	F/N	F/N [140]
*Hd16/EL1*	LOC_Os03g57940	Os03g0793500	F/W	F/W [114]
*ELF3/Hd17/EF7*	LOC_Os06g05060	Os06g0142600	F/W	F/W [104]
*RFT1*	LOC_Os06g06300	Os06g0157500	F/N	F/N [133]
*Hd3a*	LOC_Os06g06320	Os06g0157700	F/N	F/W ^a^
*Hd1*	LOC_Os06g16370	Os06g0275000	F/N	F/N [126]
*Ghd7/Hd4*	LOC_Os07g15770	Os07g0261200	F/N	F [126]
*Ghd7.1/Hd2*/*OsPRR37*/*DTH7*	LOC_Os07g49460	Os07g0695100	F/N	F [100]
*Ghd8/Hd5/DTH8*	LOC_Os08g07740	Os08g0174500	F/N	F [126]
*Ehd1*	LOC_Os10g32600	Os10g0463400	F/N	Unknown
*Hd18*	LOC_Os08g04780	Os08g0143300	F/W	Unknown

a. the wild rice data query from http://ecogems.ncpgr.cn/ with 602942292 and 602942293 sites; MSU_LOC: LOC number from MSU; RAP_LOC: LOC number from RAP; F: Functional; N: Non-functional; W: Weak functional.
